# Supplementary material for: Getting More Out of Biomedical Documents with GATE's Full Lifecycle Open Source Text Analytics
Source: PLoS Comput Biol. 2013 Feb 7;9(2):e1002854. doi: 10.1371/journal.pcbi.1002854 (PMC3567135; doi:10.1371/journal.pcbi.1002854)
Supplement: Dataset S1 — GATE software. Dataset S1 bundled with this paper contains a distribution of GATE (or see http://gate.ac.uk/download/). (TGZ) [file pcbi.1002854.s001.tgz › plos-gate/index.html]

GATE - a General Architecture for Text Engineering


## GATE documentation

Up-to-date versions of the documentation are available at
http://gate.ac.uk/:

- the software documentation- the User Guide (HTML)

Local copies:

- the software documentation- the User Guide (PDF)

## GATE licencing

This software is licenced under the
GNU Library General Public Licence.
